# Computational screening of antimicrobial peptides for *Acinetobacter baumannii*

**DOI:** 10.1371/journal.pone.0219693

**Published:** 2019-10-02

**Authors:** Ayan Majumder, Malay Ranjan Biswal, Meher K. Prakash

**Affiliations:** Theoretical Science Unit, Jawaharlal Nehru Centre for Advanced Scientific Research, Jakkur, Bengaluru, India; Academia Sinica, TAIWAN

## Abstract

*Acinetobacter baumannii*, has been developing resistance to even the last line of drugs. Antimicrobial peptides (AMPs) to which bacteria do not develop resistance easily may be the last hope. A few independent experimental studies have designed and studied the activity of AMPs on *A. baumannii*, however the number of such studies are still limited. With the goal of developing a rational approach to the screening of AMPs against *A. baumannii*, we carefully curated the drug activity data from 75 cationic AMPs, all measured with a similar protocol, and on the same ATCC 19606 strain. A quantitative model developed and validated with a part of the data. While the model may be used for predicting the activity of any designed AMPs, in this work, we perform an *in silico* screening for the entire database of naturally occurring AMPs, to provide a rational guidance in this urgently needed drug development.

## Introduction

*Acinetobacter baumannii* (*A. baumannii*) [1] is mainly implicated in hospital infections and is responsible for 80% of the *Acinetobacter* infections. *A. baumannii* can also be found on normal human skin, but it generally does not pose a threat to a healthy person [2–5], besides the not-so-frequent skin and soft tissue infections, infections in the surgical site, urinary tract infection, etc [6, 7]. In the past 30 years, *A. baumannii* has evolved into a multidrug resistant (MDR) [8–10] opportunistic pathogen that selectively infects seriously ill patients in intensive care unit (ICU), trauma or burn patients [2–4, 11]. The presence of intrinsic efflux pump and high rates of genetic adaptation, contributes to adaptation against the antibiotics [12–14]. Besides, it also possesses several beta-lactamase genes which offer resistance against beta-lactam antibiotics [15, 16]. *A. baumannii* has also been developing resistance against carbapenem [17] which had been one of the last line of drugs against it. Combination therapies such as of colistin, polymixin B, and tigecycline are used to treat MDR strains, but these are complex compared to a single drug when it comes to quantification of the effect and the validation of their safety [18–20]. Due to the growing concern about MDR, new types of antimicrobial agents are needed.

Antimicrobial peptides (AMP) are a fundamental part of the innate defense system and are reportedly present in organisms from bacteria and fungus to humans [21, 22]. Although several modes of AMP activity, including DNA damage [23], RNA damage [24] and targeting ribosomes [25–27] regulatory enzymes [28] or other proteins [29] have been proposed, it is generally believed that the positively charged AMPs act by disrupting the bacterial membrane [30–32] and the membrane disruption is one of the key factor for the AMP activity [29, 33, 34]. Because of this fundamental difference in the mechanism compared to the traditional drugs, it is believed that the bacteria do not develop resistance easily against AMPs [21]. The low toxicity of AMPs towards human cells and their tendency not to result in resistant strains makes them an ideal rational choice as the next generation antimicrobial agents [35–37], possibly eventually becoming effective drugs for *A. baumannii*.

Quantitative Structure and Activity Relationship (QSAR) [38] is an approach in computer aided rational drug design, which uses biophysical or biochemical parameters of the molecules to develop a quantitative relation with the measured activities. Once validated, the computational model can be used for predicting the activities of the possible drug candidates and for pre-screening them. Recent studies have developed a QSAR relation using 29 small molecule drug candidates which act on the oxphos metabolic path of *A. baumannii* [39]. As noted above, since bacteria are less likely to develop resistance against AMP based drugs, we focus on QSAR for AMPs against *A. baumannii*.

The present work has three major objectives. Several experimental groups have independently evaluated the activity of AMP against *A. baumannii*. We curated these experimental results against a single, well studied target, ATCC 19606 strain, whose activity is quantified using Clinical and Laboratory Standards Institute (CLSI) or related protocols. [40] We developed a computational model using neural networks to rationally predict the activity from the biochemical attributes of the AMP. Since *A. baumannii* is a growing threat, while realizing the potential limitations of training on 75 peptides, we also predict the activity of all the naturally occurring AMPs in the AMP database to enable a rational screening of AMPs against *A. baumannii*.

## Methods

### Curation of data

Training QSAR models with data from multiple sources, obtained with different protocols and on different strains can lead to poor predictive capabilities. [41] In order to standardize the data used in the analysis, we used three criteria for inclusion- the tests should be on ATCC 19606 strain, with cationic antimicrobial peptides and studied according to the CLSI or equivalent guidelines. With these inclusion criteria, we believed that the mechanism of antibiotic action will be similar and the data curated from different sources can be compared. Since data availability was limited, we had to include data from different groups. AMP sequence and activity data against *A. Baumannii* was curated from different sources [42–52] and is presented in **Table A in** S1 File. The curated AMP data set had the activity of 75 AMPs with their length ranging from 10 to 43 amino acids and charges in the range +1 to +12. Of these, for 63 AMPs the MIC was available (referred to as quantitative data), and for the remaining 12, only the lower bound of minimum inhibitory concentration (MIC) (refered to as the qualitative data).

### Parameter computation

*in vivo* aggregation propensity is calculated by using a web-based software AGGRESCAN [53]. Where the aggregation propensity is calculated on the basis of aggregation- propensity scale of amino acids. *in vitro* aggregation propensity is calculated by using TANGO software (with ionic strength 0.02M, pH 7.0 and T = 298K) [54], where we only consider the *β*-sheet aggregation term. Aliphatic index of the peptides is calculated as described by Ikai [55]. Grand average hydropathy is calculated on the scale given by Kyte-Doolittle [56] and the hydrophobic moment is calculated by using HELIQUEST software [57]. The toxicity of the AMPs was predicted using *ToxinPred* (http://crdd.osdd.net/raghava/toxinpred/). [58] The method allows for the prediction of toxicity of peptides shorter than 50 amino acids. However, this was not a limitation as peptides longer than that are anyways complicated to synthesize and may not be ideal drug candidates.

### Artificial neural network

Since the available data is limited, we used used both the quantitative and the qualitative data, albeit with different proportions, to train and test the models. We used 63 of the MIC values from the quantitative data and 3 from the qualitative data for which the cited lower bound was treated as the MIC for the purpose of this analysis. We performed a 10-fold cross validation to check the robustness of our models. To do the 10-fold cross validation, we divide the data set into 10 different test sets, each contains 7 data points. We performed the artificial neural network (ANN) calculation for each test set by taking 53 data points for training and 6 data points for validation. Rest of the 9 points from the qualitative data are used for an independent qualitative test. The activity of the AMPs was predicted by ANN model with an open module for machine learning called Scikit-learn [59] in Python. For the activation function, logistic function was used and low memory BFGS optimization algorithm was used a solver. Three independent neural network calculations have been performed to do the 10-fold cross validation, by using a hidden layer of 6 neurons, 8 neurons and 10 neurons. 2500 trial runs in each case were made by taking 50 different random initializations for the input biases and 50 random choices for the training and validation sets. We screened the results of these 2500 trials with $R_{training}^{2}>0.7$ and $R_{validation}^{2}>0.6$. Two best models were selected based on the result obtained from the 10-fold cross validation. The models were expected to perform with $R_{test}^{2}>0.8$ for the quantitative data and at least 5 predictions for the qualitative data set. These models were then used to predict the MIC values of a complete AMP database [60–62] (https://aps.unmc.edu).

## Results

### Curated data for AMPs and their effectiveness

**Fig 1:**
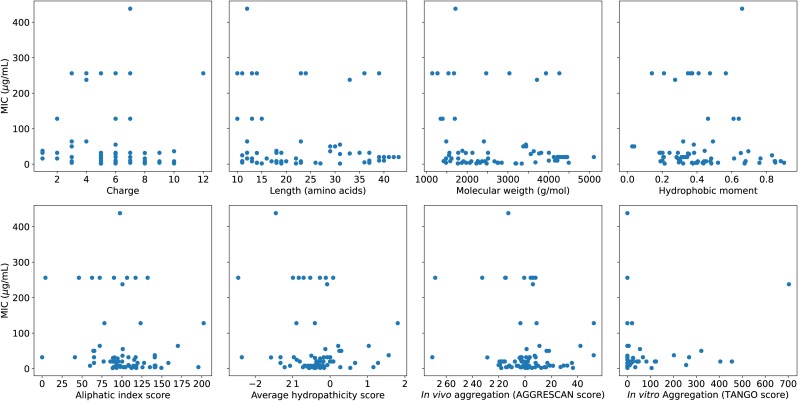
MIC versus different parameters. The AMPs used in the analysis along with the sequences and biophysical parameters are given in Table A in S1 File.

The data on the activity of AMPs on *A. baumannii* is scattered in literature. We curated the data mainly with the goal of developing a quantitative model, and hence restricted the focus to the most commonly studied ATCC 19606 strain. To maintain uniformity of standards, we included studies which were performed according to CLSI or equivalent guidelines. The sequence data and the antimicrobial activity of these peptides measured as the MIC was gathered (**Table A in** S1 File). Overall, the comprehensive collection of the data on AMP activity allowed a classification based on the various biophysical parameters which are commonly used for developing a quantitative relation with activity: (1) charge, which draws the AMPs selectively to anionic membrane, (2) length, reflecting how it has to be commensurate with the membrane thickness for an improved activity [63, 64] (3) molecular weight, which gives an idea of the bulkiness and membrane penetration efficiency (4) hydrophobic moment (*μ*_*H*_), [57] which quantifies the amphipathic characters required to form pores in the membrane, (5) aliphatic index [55], which indicates the volume of aliphatic content (A, V, I and L) of the peptide, (6) grand average of hydropathy (GRAVY) based on Kyte-Doolittle hydropathicity scale [56], (7) *in vivo* aggregation propensity, calculated by using a web-based software AGGRESCAN [53] and (8) *in vitro* (*β*-sheet) aggregation propensity, calculated by using TANGO software (with ionic strength 0.02 M, pH 7.0 and temperature 298 K) [54]. The *in vitro* aggregation, before interaction with the membrane can at times stop proteolytic degradation [65] by the bacteria but in many other cases reduce the drug potency [66, 67]. Further, the aggregation propensity affects the barrel-stave [68] and carpet mechanisms [63] of action differently. Toxicity of peptides obtained from ToxinPred [58] was categorical, and it was used only to classify the AMPs from the database as potential drug candidates, and not for the activity prediction. The distribution of the eight parameters for all the curated AMPs are given in **Fig A in** S1 File and their individual relation with MIC in Fig 1, which shows that each of the parameters individually is not sufficient to describe the activity.

### Quantitative models for AMP activity

ANN model was used to obtain the relationship between the various above-mentioned parameters and MIC values (**Methods**). A schematic of how we developed the model is shown in **Fig B in** S1 File. The first step was to create a model with the activity data from 75 AMPs, of which some were used for an internal assessment of the quality of predictions. The second step was to use the test set in the 75 AMP data analysis as a secondary validation for refining the choice of model that can be used for making the predictions for the AMP database. The details are as follows. Out of the 75 AMPs curated, for 12 of them a lower bound of MIC, as being greater than a certain value (**Table B in** S1 File), rather than a precise number was cited. To include them in the analysis, and not to reduce the data size which is already small (75 AMPs), we created two independent test sets, one in which a quantitative MIC comparison was made (referred to as quantitative data) and another qualitative one in which the calculated MIC was checked if it was more than the experimental lower bound (referred to as qualitative data). The combined data set with quantitative and qualitative data was used to construct training, validation and test sets (**Methods**). We performed a 10-fold cross validation with three different architectures with 6, 8 and 10 hidden neurons respectively. The overall error in the architecture with 8 neurons was optimal, thus justifying a small sampling around it with 6 and 10 neurons (**Table C in** S1 File). However, all three architectures were satisfactory in their predictions (**Figs C**, **D and E respectively in** S1 File), resulting in many models, which qualify for the criteria ($R_{training}^{2}>\;0.7$ & $R_{validation}^{2}>\;0.6$). Several of these models also had good predictions for the test sets, which are about 10% of the data.

### Selecting the best model

**Fig 2:**
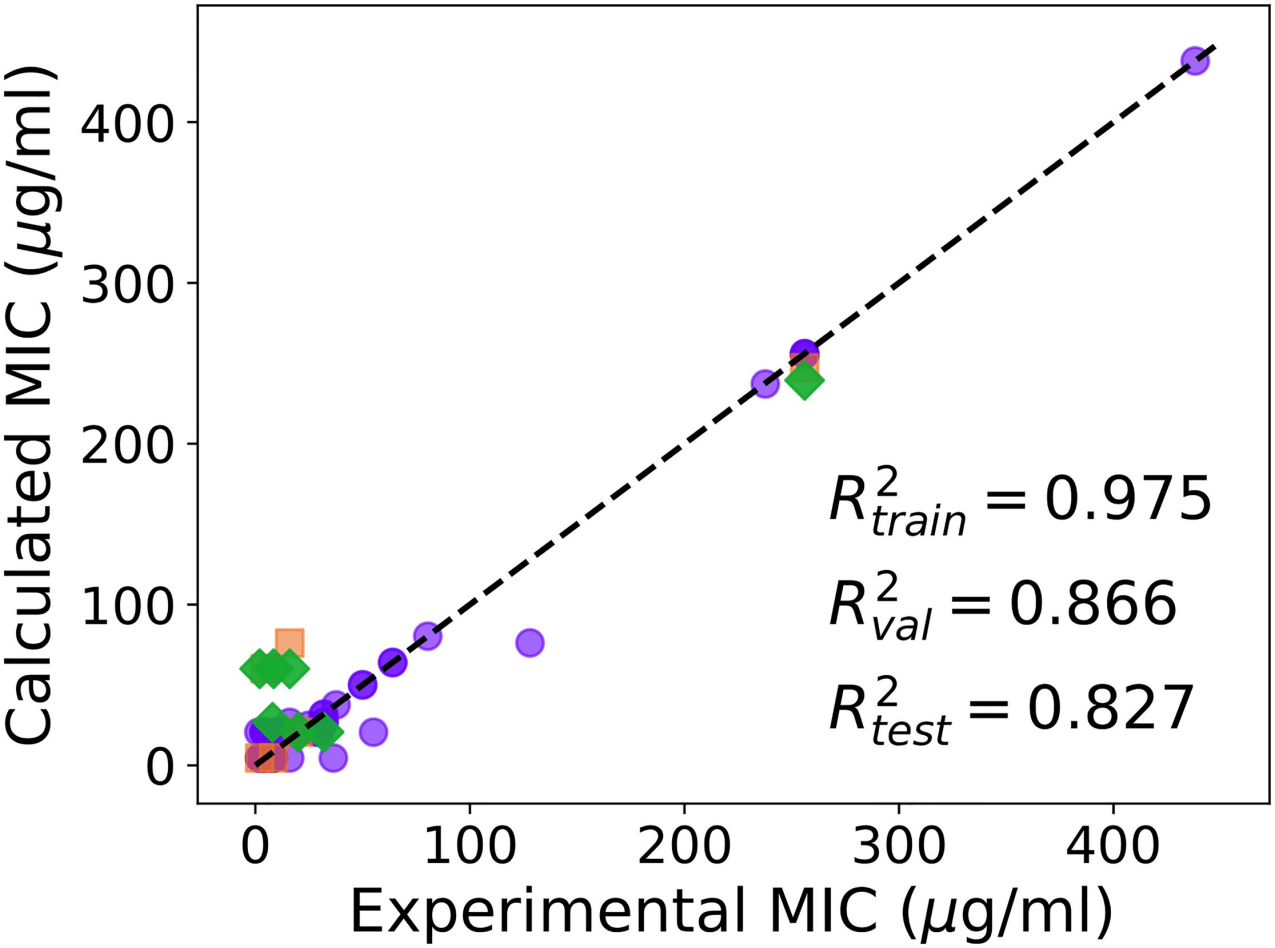
Comparison of the experimental and calculated MIC (*μ*g/ml) of curated AMPs on *A. baumannii* obtained from Model-1, calculated by using 8 hidden neurons. Training (purple circles), validation (orange squares) and test (green diamonds) sets are shown. The data used in the analysis is shown in Table 1 in S1 File.

In a traditional QSAR analysis, the choice of the best model would be guided by the combination of the best $R_{training}^{2}$ and $R_{validation}^{2}$, following which $R_{test}^{2}$ on a small fraction of the data, in our case 7 data points, comes as a consequence. Since the goal of screening through the large set of potential AMPs whose activities against an extremely important pathogen are not yet available is more ambitious than performing well on these 7 points, we performed a secondary validation check to select the best models. We used two additional criteria: $R_{test}^{2}>0.6$ for the quantitative and that at least 5 predictions in qualitative data set were correct to within a factor of 2 (**Table B in** S1 File). Two models satisfied these conditions, with $R_{test}^{2}>0.8$ and they were selected. The best among these models (referred to as Model-1) obtained from the calculation with 8 hidden neurons, had good predictions ($R_{training}^{2}=0.975$, $R_{validation}^{2}=0.866$ and $R_{test}^{2}=0.827$). The experimental MIC for the quantitative data set versus MIC values predicted from Model-1 is shown in Fig 2. Results obtained from another model (Model-2) are given in **Fig F in** S1 File.

### Predicting the results for naturally occurring AMPs

Considering the health threat *A. baumannii* is posing, and the potential of AMPs for antibiotic-resistance-free activity, we propose a rational basis for an *in silico* screening of AMPs active against *A. baumannii*. Our models were used to predict the MIC values of the 2338 AMPs obtained from database [60–62] (https://aps.unmc.edu) of naturally occurring AMPs. We made the predictions from Model-1 and Model-2 (S2 File). In order to reduce the risk of a poorly trained ANN model with limited data, we filtered these results for a consistent prediction that is within ΔMIC ≤ 5 *μ*g/ml for both the models (Table 1). Despite the potential statistical limitations of training and validating on 75 AMPs, a pre-screening to rationally sort multiple AMPs with their predicted activity, *in vitro* and *in vivo* aggregation potential, toxicity and length (a surrogate for synthetic complexity), all are provided in Table 1 and in the S2 File. The computational scripts and the predictions are made accessible (S3 File), to provide an immediate access to a pool of rational choices that can help progress towards large scale experimental testing, considering the extreme urgency of developing effective strategies to combat the superbug, *A. baumannii*

**Table 1:** Using the 2 different models, we predicted the activity of 2338 naturally occurring AMPs documented in the AMP database. The complete list of predictions are given in the S2 File. However, of these the AMPs which had consistent predictions from both the models (ΔMIC ≤ 5 *μ*g/ml) were selected and presented in this table. All of these were peptides listed below were non-toxic according to the predictions from ToxinPred (http://crdd.osdd.net/raghava/toxinpred/) [58].

Peptide	Sequence	Length	Model-1 MIC(*μ*g/ml)	Model-2 MIC(*μ*g/ml)
AP01466	VNWKKILGKIIKVAK	15	2.84	6.20
AP00143	KKLLKWLKKLL	11	9.08	4.59
AP01456	VGKTWIKVIRGIGKSKIKWQ	20	9.34	4.60
AP00708	GFKRIVQRIKDFLRNLV	17	9.38	4.59
AP00161	GLWSKIKTAGKSVAKAAAKAAVKAVTNAV	29	14.24	10.44
AP00577	GLFTLIKGAAKLIGKTVAKEAGKTGLELMACKITNQC	37	14.24	15.64
AP00608	KRIVQRIKDFLR	12	14.40	14.25
AP01525	SWLSKTYKKLENSAKKRISEGIAIAIQGGPR	31	16.38	20.78
AP00869	ILPLVGNLLNDLL	13	17.60	20.67
AP00425	GCWSTVLGGLKKFAKGGLEAIVNPK	25	18.23	20.86
AP01388	GLLSGILNSAGGLLGNLIGSLSN	23	21.02	20.67
AP00733	LLGDFFRKAREKIGEEFKRIVQRIKDFLRNLVPRTES	37	21.70	19.43
AP01387	GLLSGILNTAGGLLGNLIGSLSN	23	22.83	20.67
AP00061	GIGGVLLSAGKAALKGLAKVLAEKYAN	27	23.57	20.66
AP00210	GMASKAGAIAGKIAKVALKAL	21	25.07	20.26
AP00006	GNNRPVYIPQPRPPHPRI	18	27.10	26.77
AP00007	GNNRPVYIPQPRPPHPRL	18	27.10	26.77
AP00024	GVSGHGQHGVHG	12	27.10	27.98
AP00025	HGVSGHGQHGVHG	13	27.10	26.77
AP00141	RKKWFW	6	27.10	26.77
AP00150	ILPWKWPWWPWRR	13	27.10	26.77
AP00152	VRRFPWWWPFLRR	13	27.10	26.77
AP00169	GRPNPVNTKPTPYPRL	16	27.10	26.77
AP00170	VDKGSYLPRPTPPRPIYNRN	20	27.10	26.77
AP00172	GKPRPYSPRPTSHPRPIRV	19	27.10	26.79
AP00190	HPLKQYWWRPSI	12	27.10	26.77
AP00191	ECRRLCYKQRCVTYCRGR	18	27.10	26.77
AP00211	RRWCFRVCYRGFCYRKCR	18	27.10	26.77
AP00212	RRWCFRVCYKGFCYRKCR	18	27.10	26.77
AP00213	KWCFRVCYRGICYRKCR	17	27.10	26.77

### Parameter importance in model

**Fig 3:**
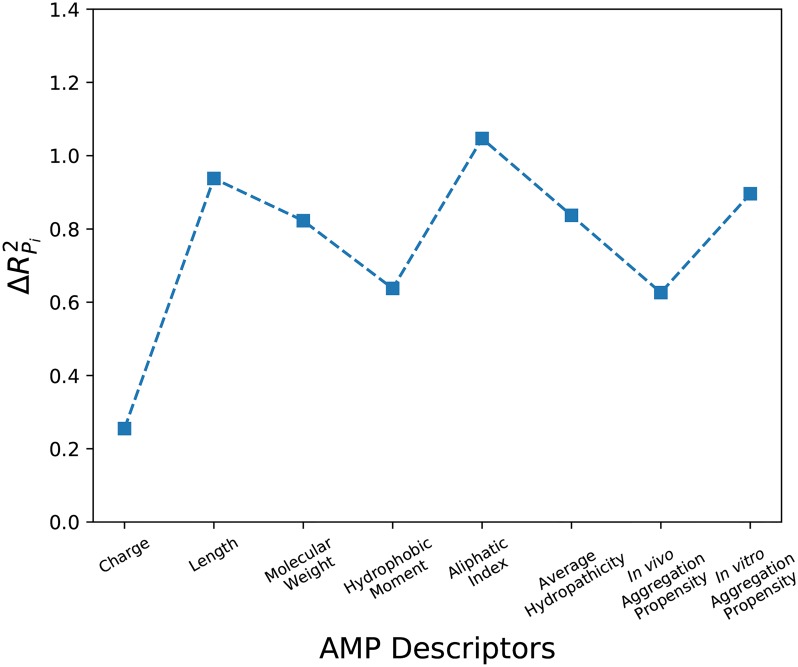
The relative importance of the different parameters in Model-1 is shown. Aliphatic index influences the outcomes of the predictions the most in this model.

It is important to know which are the parameters (*P*_*i*_) that are most responsible for the activity on *A. baumannii*. In the combined training and validation set used for accepting the models, we replaced (*P*_*i*_) with its average <*P*_*i*_> and measure the difference $\Delta R_{P_{i}}^{2}=R_{training+validation}^{2}-R_{training+validation,<P_{i}>}^{2}$. $\Delta R_{P_{i}}^{2}$ is treated as reflecting the importance of the parameter. The results obtained from Model-1 are given in Fig 3 and the result obtained from another model is given in **Fig G in** S1 File. From our calculations, we found out that the aliphatic index is the most important parameter in both the models.

### Relevance of predictions for MDR strains

In order to reduce the uncertainties, our computational model was trained on data standardized in three ways, *A. baumannii* strain used, choice of cationic AMPs and measurements by CLSI method. However, considering the threat that *A. baumannii* MDR strains are posing, it is important to ask whether our calculations have any relevance to these clinical variants. The two limitations of this work are the smaller data size used for training, and it was based on ATCC 19606 strain. Interestingly, in the limited studies that we found the activity of cationic AMPs against ATCC 19606 and other MDR strains of *A. baumannii* are comparable [43, 46], thus potentially removing the latter strain specific data limitation for *A. baumannii*, although for other bacteria, such as *S. aureus* the activity changes quite significantly with the strain [69, 70]. Drawing confidence from this fact, we used our models to predict the activity for a few MDR strains [71–73]. The results reported in **Table D in** S1 File are encouraging at this stage, although more such validations will be helpful in establishing the utility of the screening models we proposed.

### Conclusions

To our knowledge, the present work is the only QSAR study for predicting AMP activity against *A. baumannii*. The present work is different from the only other QSAR in two different ways, using AMPs instead of small molecules for a better tolerance to antibiotic resistance and a slightly larger set (75 AMPs compared to 29 small molecules). Using the ANN models we developed, we could make quantitative predictions for the entire database of naturally occuring AMPs. We hope that our work will inspire the further studies quantifying the activity of AMPs on *A. baumannii*, some of which may follow the activity predictions and others that differ offer an opportunity to retrain the ANN models.

## Supporting information

S1 File S1 File contains **Table A**, details of 75 curated AMPs, **Fig A**, histogram of all parameters corresponding to AMPs, **Table B**, experimental and predicted MIC values of 9 qualitative data that were used for additional test, **Table C**, 10 fold cross validation analysis with different number of hidden neurons, **Fig B**, **Fig C**, **Fig D**, comparison of experimental and predicted MIC obtained from 10 fold cross validation using 6, 8 and 10 neurons respectively, **Fig E**, comparison of experimental and predicted MIC for Model-2, calculated using 6 neurons, **Fig F**, importance of different parameters used in Model-2, **Fig G**, flow chart showing the schematic of how the ANN model were developed, **Table D**, comparison of the experimental and predicted MIC values for the MDR *A. baumannii* strains.
(PDF)

S2 File S2 File contains two data sheets in xlsx format. These contains the prediction for the 75 different curated peptides and prediction for the 2338 natural occurring AMPs.
(XLSX)

S3 File ZIP file contains python script and data file for predicting antimicrobial activity against *A. baumannii*. (ZIP)
