# Supplementary material for: Computational screening of antimicrobial peptides for Acinetobacter baumannii
Source: PLoS One. 2019 Oct 2;14(10):e0219693. doi: 10.1371/journal.pone.0219693 (PMC6774513; doi:10.1371/journal.pone.0219693)
Supplement: S1 File — contains Table A, details of 75 curated AMPs, Fig A, histogram of all parameters corresponding to AMPs, Table B, experimental and predicted MIC values of 9 qualitative data that were used for additional test, Table C, 10 fold cross validation analysis with different number of hidden neurons, Fig B, Fig C, Fig D, comparison of experimental and predicted MIC obtained from 10 fold cross validation using 6, 8 and 10 neurons respectively, Fig E, comparison of experimental and predicted MIC for Model-2, calculated using 6 neurons, Fig F, importance of different parameters used in Model-2, Fig G, flow chart showing the schematic of how the ANN model were developed, Table D, comparison of the experimental and predicted MIC values for the MDR A. baumannii strains. (PDF) [file pone.0219693.s001.pdf]

Supporting Information

Computational screening of antimicrobial peptides for *Acinetobacter baumannii*

Ayan Majumder[1¶], Malay Ranjan Biswal[1¶], Meher K. Prakash[1*]

1 Theoretical Science Unit, Jawaharlal Nehru Centre for Advanced Scientific Research, Jakkur, Bengaluru, India

¶These authors contributed equally to this work.
* meher@jncasr.ac.in

1 Supplementary Table A

Table A. Details of all the 75 antimicrobial peptides and their experimental protocol used in the present work. Qualitative test set is indicated by blue color, and with a lower bound in MIC.

Sl. No	Peptide	Sequence	MIC (μg/ml)	References	Methods
1	Bactenicin	RLCRIVVIRVCR	64	[1]	CLSI
2	Cecropin A	KWKLFKKIEKVGQNIRDGIIKAGPAVAVVGQATQIAK	32	[1]	CLSI
3	Cecropin B	KWKIFKKIEKVGRNIRNGIIKAGPAVAVLGEAKAL	32	[1]	CLSI
4	Cecropin P1	SWLSKTAKKLENSAKKRISEGIAIAIQGGPR	1.6	[1]	CLSI
5	Histatin 8	KFHEKHHSHRGY	32	[1]	CLSI
6	HNP-1	ACYCRIPACIAGERRYGTCIYQGRLWAFCC	50	[1]	CLSI
7	HNP-2	CYCRIPACIAGERRYGTCIYQGRLWAFCC	50	[1]	CLSI
8	Indolicidin	ILPWKWPWWPWRR	8	[1]	CLSI
9	Magainin I	GIGKFLHSAGKFGKAFVGEIMKS	64	[1]	CLSI
10	Magainin II	GIGKFLHSAKKFGKAFVGEIMNS	256	[1]	CLSI
11	Mastoparan	INLKALAALAKKIL	4	[1]	CLSI
12	Melittin	GIGAVLKVLTTGLPALISWIKRKRQQ	4	[1]	CLSI
13	β-Defensin	DHYNCVSSGGQCLYSACPIFTKIQGTCYRGKAKCCK	256	[1]	CLSI
14	LysAB2 P0[1]	NPEKALEPLIAIQIAIKGMLNGWFTGVGFRRKR	237.66	[2]	Conlon et.al. [3]
15	LysAB2 P1[1]	EKALEKLIAIQKAIKGMLNGWFTGVGFRRKR	28.48	[2]	Conlon et.al. [3]
16	LysAB2 P2[1]	EKALEKLIAIQKAIKGMLAGWFTGVGARRKR	55.06	[2]	Conlon et.al. [3]
17	LysAB2 P3[1]	NPEKALEKLIAIQKAIKGMLNGWFTGVGFRRKR	30.12	[2]	Conlon et.al. [3]
18	LL-37	LLGDFFRKSKEKIGKEFKRIVQRIKDFLRNLVPRTES	4	[4]	CLSI
19	Aurein 1.2	GLFDIIKKIAESF -NH_2	16	[4]	CLSI
20	CAMEL	KWKLFKKIGAVLKVL -NH_2	2	[4]	CLSI
21	Citropin 1.1	GLFDVIKKVASVIGGL -NH_2	16	[4]	CLSI
22	Omiganan	ILRWPWWPWRRK -NH_2	32	[4]	CLSI
23	r-Omiganan	KRRWPWWPWRLI -NH_2	16	[4]	CLSI
24	Pexiganan	GIGKFLKKAKKFGKAFVKILKK -NH_2	2	[4]	CLSI
25	Temporin A	FLPLIGRVLSGIL -NH_2	128	[4]	CLSI
26	rr	WLRRIKAWLRR	24.86	[5]	CLSI
27	rr2	WIRRIKKWIRRVHK	3.95	[5]	CLSI
28	rr3	WLRRIKAWLRRKRK	31.46	[5]	CLSI
29	rr4	WLRRIKAWLRRIKA	3.73	[5]	CLSI
30	CLP-19	CRKPTFRRLKWKIKFKFKC	80.36	[6]	CLSI
31	C18G[2]	ALWKKLLKKLLKSAKKLG -NH_2	3.87	[7]	Wiegand et.al. [8]
32	C18G-Arg[2]	ALWRRLLRRLLRSARRLG -NH_2	8.48	[7]	Wiegand et.al. [8]
33	NK-2	KILRGVCKKIMRTFLRRISKDILTGKK -NH_2	2	[9]	CLSI
34	NK27	KILRGVSKKIMRTFLRRISKDILTGKK -NH_2	2	[9]	CLSI
35	N17	KILRGVSKKIMRTFLRR -NH_2	8	[9]	CLSI
36	C20	KKIMRTFLRRISKDILTGKK -NH_2	8	[9]	CLSI
37	C20-DK	KKIMRTFLRRISKKILTGKK -NH_2	8	[9]	CLSI
38	NK23a	KISKKIMRTFLRRISKDILTGKK -NH_2	4	[9]	CLSI
39	NK23b	KILRGVSKKIMRRISKDILTGKK -NH_2	8	[9]	CLSI
40	NK22b	KILGVSKKIMRRISKDILTGKK -NH_2	16	[9]	CLSI
41	NK23c	KILRGVSKKIMRTFLRRILTGKK -NH_2	4	[9]	CLSI
42	NK19a	KISKKIMRTFLRRILTGKK -NH_2	4	[9]	CLSI
43	NK19b	KILRGVSKKIMRRILTGKK -NH_2	2	[9]	CLSI
44	NK19b-KR	RILRGVSRRIMRRILTGRR -NH_2	8	[9]	CLSI
45	NK13	KISKKIMRTFLRR -NH_2	256	[9]	CLSI

Sl. No	Peptide	Sequence	MIC (μg/ml)	References	Methods
46	Pepcon[2]	FLFSLIPSAIGGLISAFK	37.62	[10]	Wiegand et.al. [8]
47	BP100	KKLFKKILKYL $-NH_2$	8.5	[11]	CLSI
48	RW-BP10	RRLFRRILRWL $-NH_2$	8.5	[11]	CLSI
49	BP202	KRLFRKILKYL $-NH_2$	4.5	[11]	CLSI
50	BP203	KKLFKKILRYL $-NH_2$	8.5	[11]	CLSI
51	H4	KFKKLFKKLSPVIGKEFKRIVERIKRFLR	36.34	[12]	CLSI
52	BR001[3]	KWKLFKKIEKVGQNIRDGIIKAGPAVAVVGQATQIAK $-NH_2$	10	[13]	Jorgensen et.al. [14]
53	BR002[3]	GWLKKIGKKIERVGQHTRDATIQGLGIAQQAANVAATAR $-NH_2$	10	[13]	Jorgensen et.al. [14]
54	BR003[3]	GGLKKLGKKLEGAGKRVFNAAEKALPVVAGAKALRK	5	[13]	Jorgensen et.al. [14]
55	BR005[3]	RGFRKHFNKLVKKVKHTISETAHVAKDTAVIAGSGAAVVAAT $-NH_2$	20	[13]	Jorgensen et.al. [14]
56	BR029[3]	KWKIFKKIEKAGRNIRDGIIKAGPAVSVVGEAATIYKTG	20	[13]	Jorgensen et.al. [14]
57	BR031[3]	GWLRDFGKRIERVGQHTRDATIQAIGVAQQAANVAATVRG	20	[13]	Jorgensen et.al. [14]
58	BR032[3]	GWLKKIGKKIERVGQHTRDATIQVLGVAQQAANVGPATARG	20	[13]	Jorgensen et.al. [14]
59	BR033[3]	GWLKKIGKKIERVGQHTRDATIQTIGVAQQAANVAATLKG	20	[13]	Jorgensen et.al. [14]
60	BR034[3]	GWLKKFGKKIERVGQHTRDATIQAIGVAQQAANVAATLKG	20	[13]	Jorgensen et.al. [14]
61	BR035[3]	GWLKKIGKKIERVGQHTRDASIQAIGIAQQAANVAATARG	10	[13]	Jorgensen et.al. [14]
62	BR036[3]	GWLKKIGKKIERVGQHTRDATIQVLGVAQQAANVAATARG	20	[13]	Jorgensen et.al. [14]
63	BR037[3]	GLVKKIGKKIERVGQHTRDASIQAIGIAQQAANVAATARG	20	[13]	Jorgensen et.al. [14]
64	Buforin I	AGRGKQGGKVRAKAKTRSSRAGLQFPVGRVHRLLRKGNY	>256	[1]	CLSI
65	Histatin 5	DSHAKRHHGYKRKFHEKHHSHRGY	>256	[1]	CLSI
66	rr1	WKRRIKIWKKIR	>438.06	[5]	CLSI
67	C18G-His	ALWHHLLHHLLHSAHHLG $-NH_2$	>31.93	[7]	Wiegand et.al. [8]
68	I10	KKIMRTFLRR $-NH_2$	>128	[9]	CLSI
69	NK15	KILRGVSKRILTGKK $-NH_2$	>128	[9]	CLSI
70	NK14	KILGVSKRILTGKK $-NH_2$	>256	[9]	CLSI
71	NK11	KISKRILTGKK $-NH_2$	>256	[9]	CLSI
72	NK10	ISKRILTGKK $-NH_2$	>256	[9]	CLSI
73	BR030[3]	KWKFYKKIERVGQNIRDGIIKAGPAVQVVGQQPRYIKENRFYS	>20	[13]	Jorgensen et.al. [14]
74	BR043[3]	AGFRKRFNKLVKKVKHTIKETANVSKDVAIVAGSGVAVGAAMG	>20	[13]	Jorgensen et.al. [14]
75	BR044[3]	GFRKRFNKLVKKVKHTIKETANVSKDVAIVAGSGVAVGAAMG	>20	[13]	Jorgensen et.al. [14]

[a]These MIC values are calculated according to CLSI guideline.
[b]These MIC values are calculated according to CLSI and EUCAST guideline.
[c]MIC values obtained from this procedure is comparable with the MIC values obtained according to CLSI guideline.

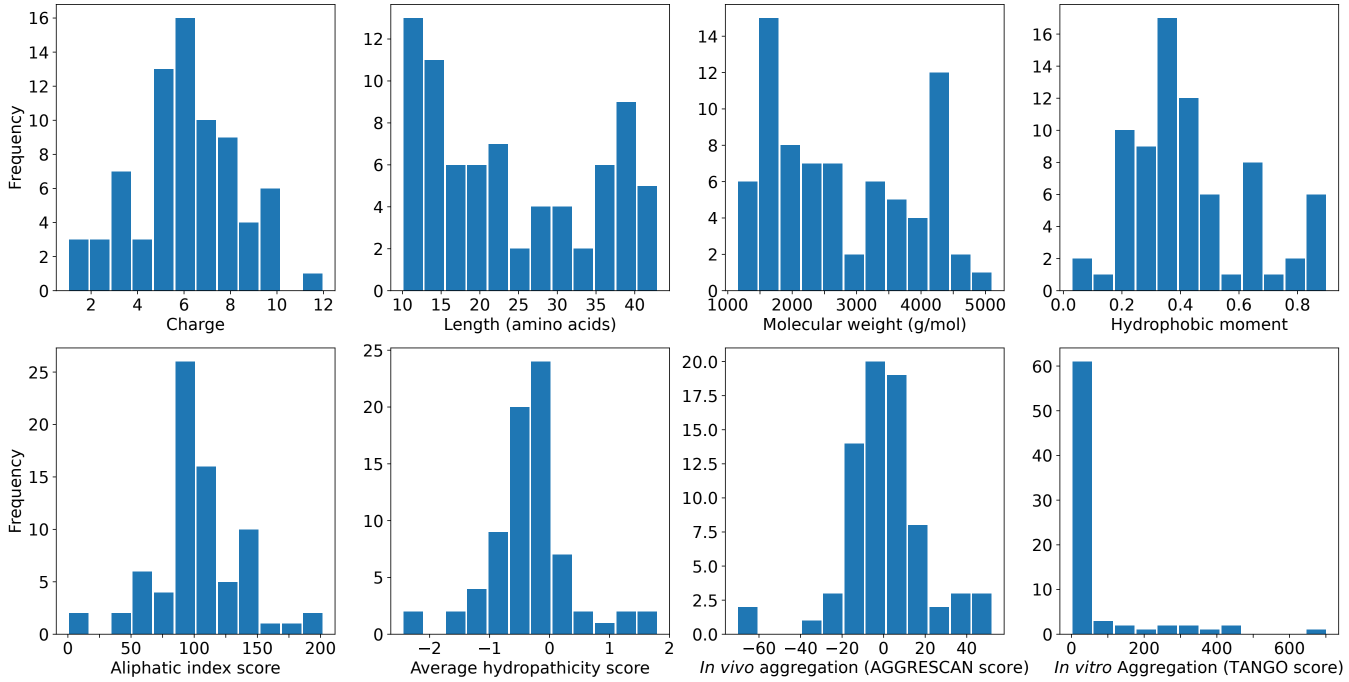

Fig A. Histogram of all the parameters corresponding to the AMPs shown in **Table A in S1 File**. The raw data of these parameters is in **S2 File**.

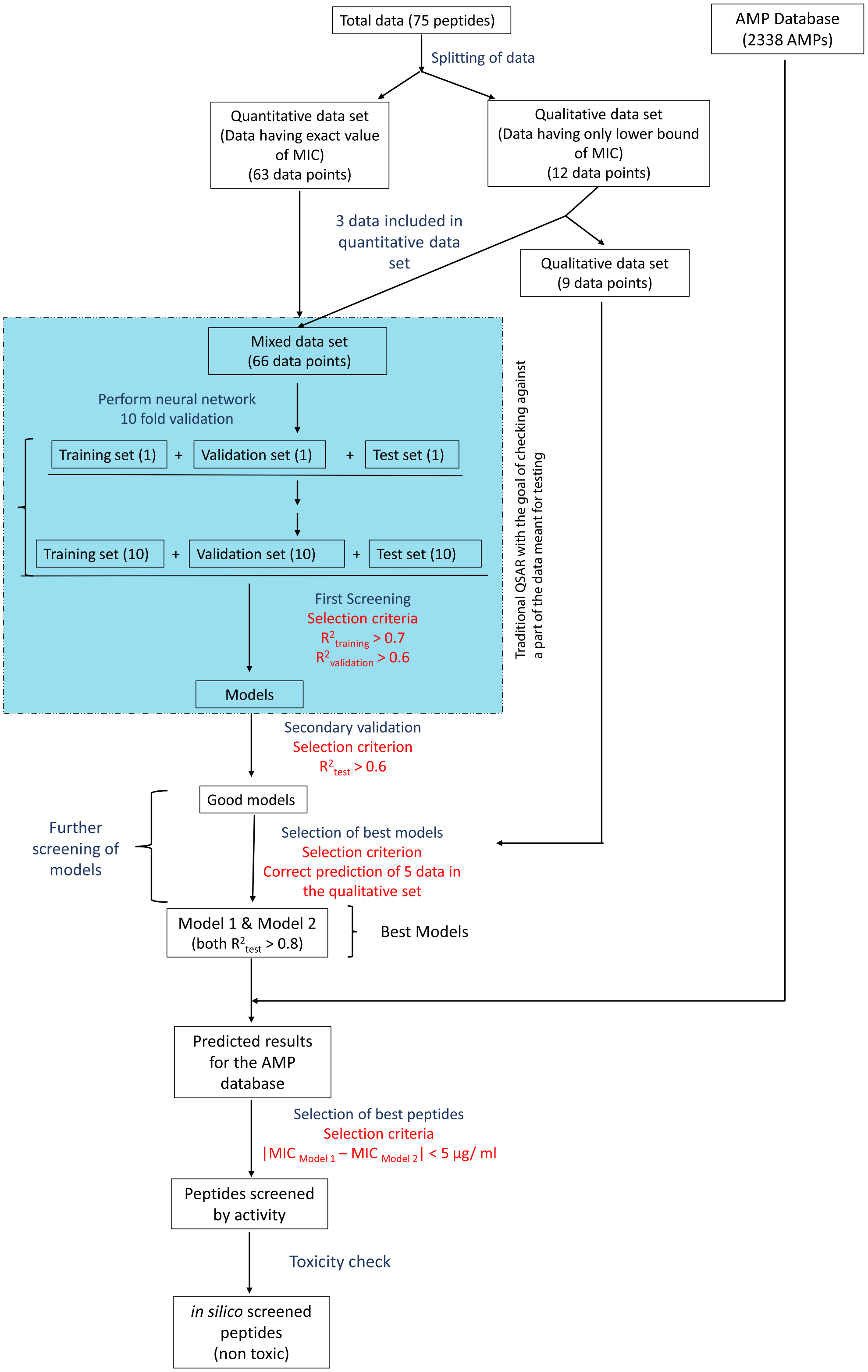

Fig B. Flow chart showing the schematic of how the ANN models were developed. The part embedded in the dotted box is the logic of selecting the best models in traditional QSAR method, if the goal had been to be content with the prediction for the test set with 7 peptides. In such methods, typically a person conducting the test or the person developing the model but chooses to stay blind to this additional test data, eventually compares the performance of the predictions on this small test set. However, in our case, the goal is to make predictions for the 2338 AMPs for which no activity measurements are yet available, a secondary validation criteria was used to screen the models further.

2 Supplementary Table B

Table B. Experimental and predicted MIC values of the 9 qualitative AMP data that were used for an additional test. Before accepting a model, at least 5 of these 9 results were verified to be more than the lower bound suggested by the experiments (to within a factor of 2). An additional condition $R^2 > 0.6$ was used with the test data set from the quantitative data.

**Name**	**Expt. MIC** μ**g/ml**	**Model-1 MIC** μ**g/ml**	**Model-2 MIC** μ**g/ml**
C18G-His	>31.9275	75.9	9.4
I10	>128	482.1	437.1
NK15	>128	4.6	14.2
NK14	>256	60.1	14.2
NK11	>256	239.17	215.1
NK10	>256	86.1	14.6
BR030	>20	255.5	214.8
BR043	>20	22.4	14.2
BR044	>20	62.6	14.2

3 Supplementary Table C

Table C. 10-fold cross validation analysis was performed with a hidden layer between the input and output layers. The hidden layer architecture with 6, 8, and 10 neurons respectively were independently modelled. Here we tabulated the number of times, out of 10, $R^2_{test} > 0.6$, as well as the mean squared error (MSE) and standard deviation (SD). The overall error was optimal with the choice of 8 neurons, although all three architectures performed satisfactorily.

	**Number of neurons in the hidden layer**		
	6 neurons	8 neurons	10 neurons
$\#R^2_{test} > 0.6$	6	9	8
MSE	1963.979	1119.023	1423.350
SD	1984.295	1112.944	1972.955

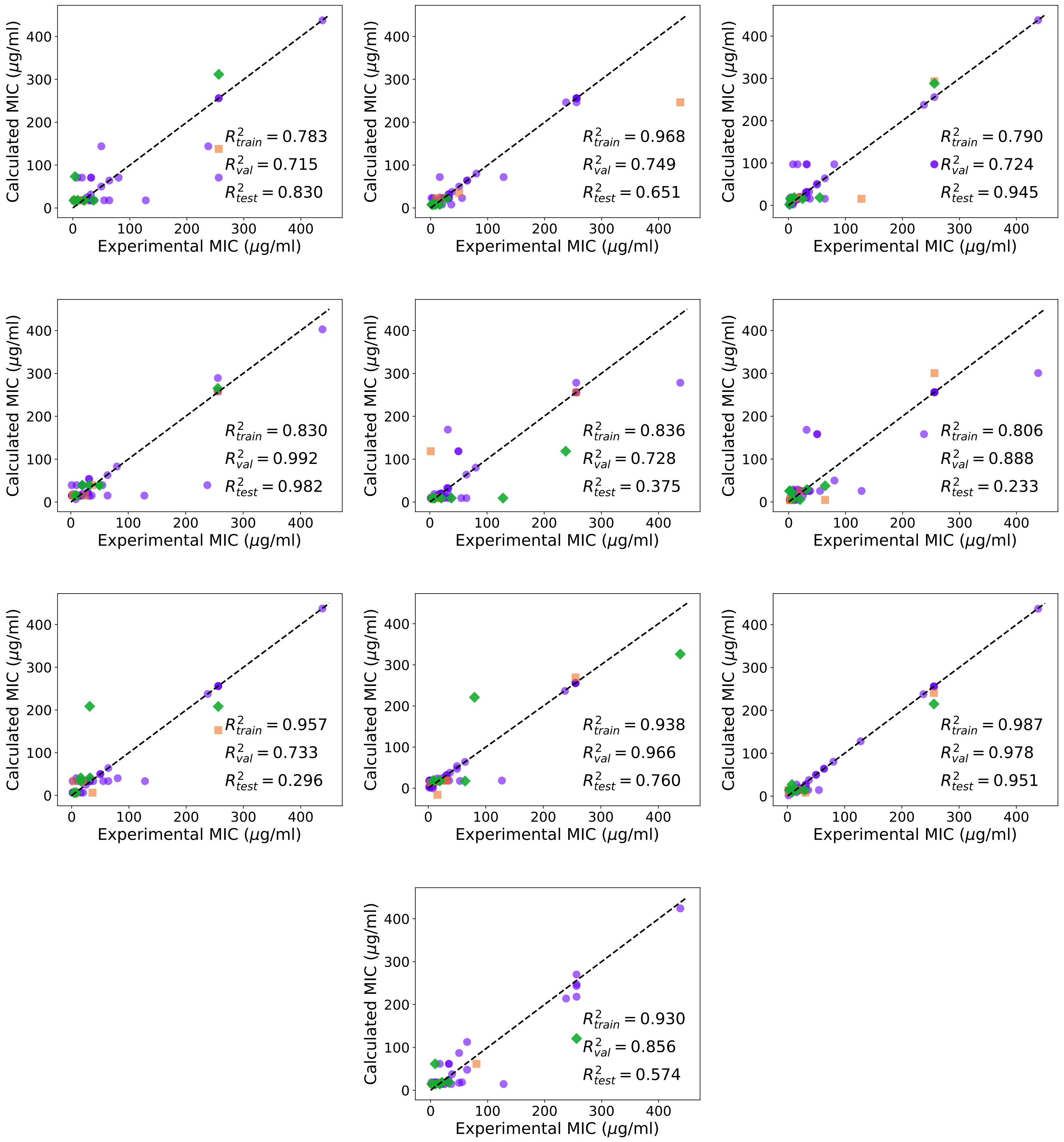

Fig C. Comparison of the experimental MIC (μg/ml) and predicted MIC (μg/ml) values of AMP obtained from the 10 fold cross validation calculation. The neural network is trained with one hidden layer consisting of 6 neurons.

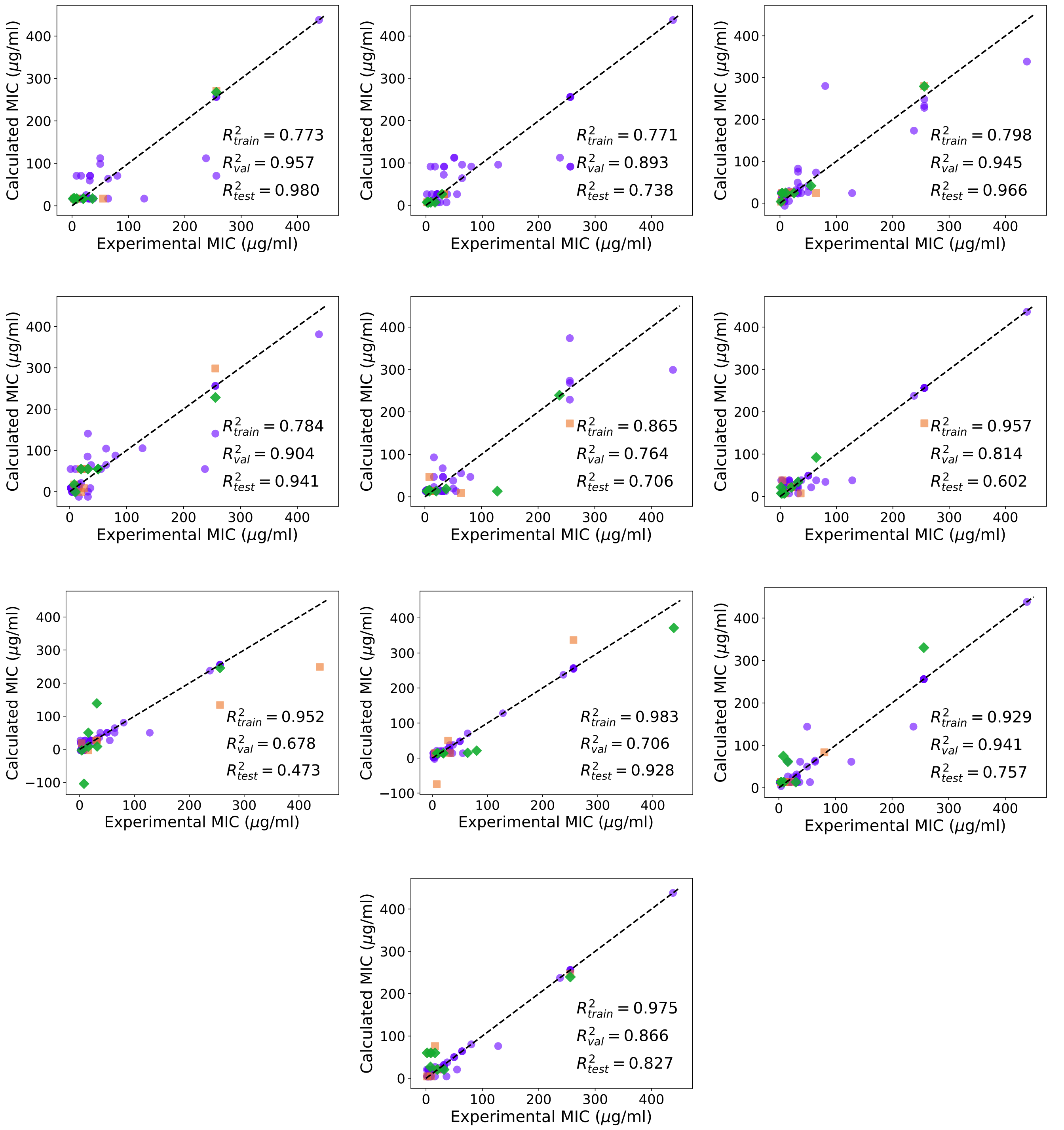

Fig D. Comparison of the experimental MIC (μg/ml) and predicted MIC (μg/ml) values of AMP obtained from the 10 fold cross validation calculation. The neural network is trained with one hidden layer consisting of 8 neurons.

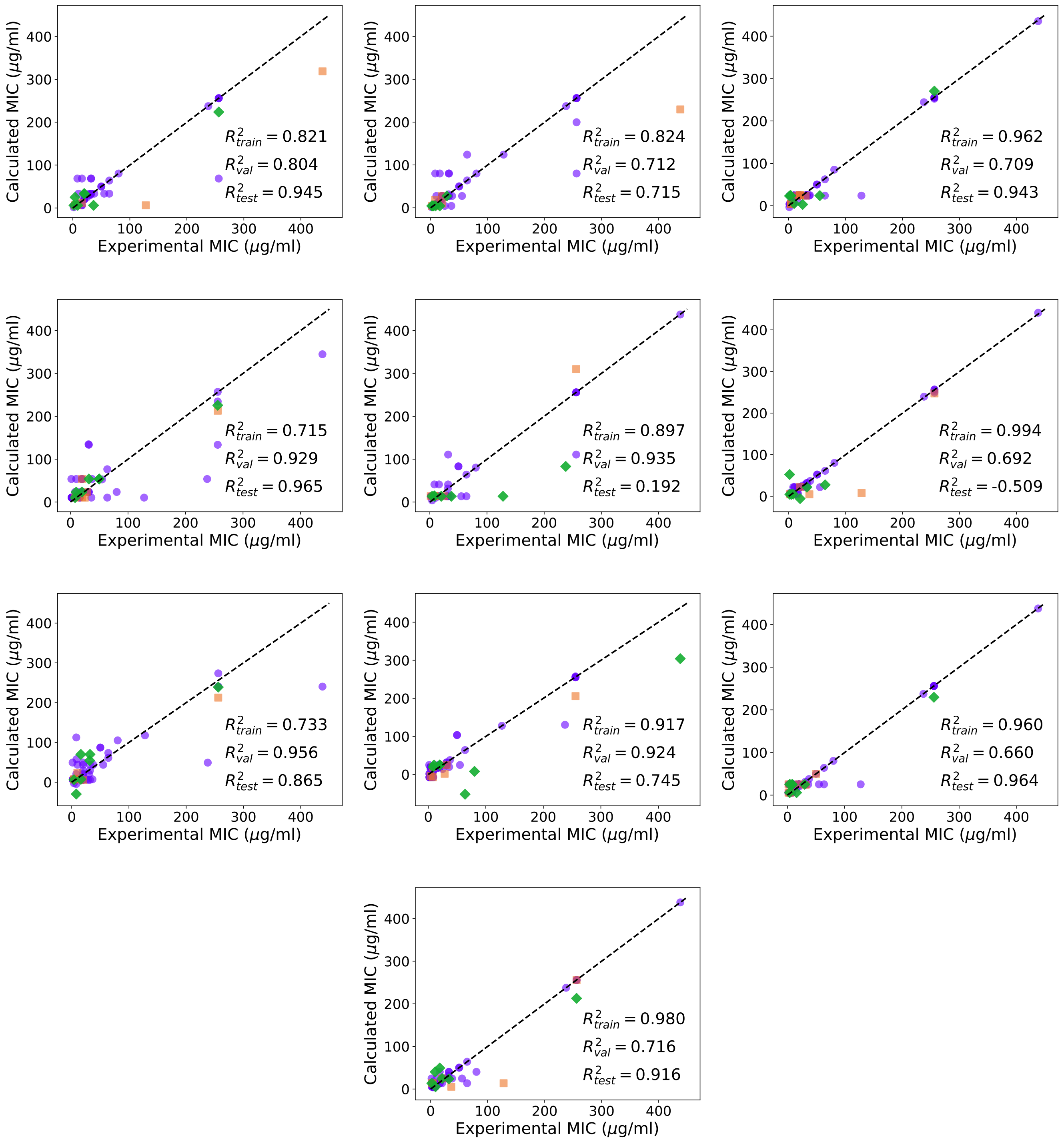

Fig E. Comparison of the experimental MIC (μg/ml) and predicted MIC (μg/ml) values of AMP obtained from the 10 fold cross validation calculation. The neural network is trained with one hidden layer consisting of 10 neurons.

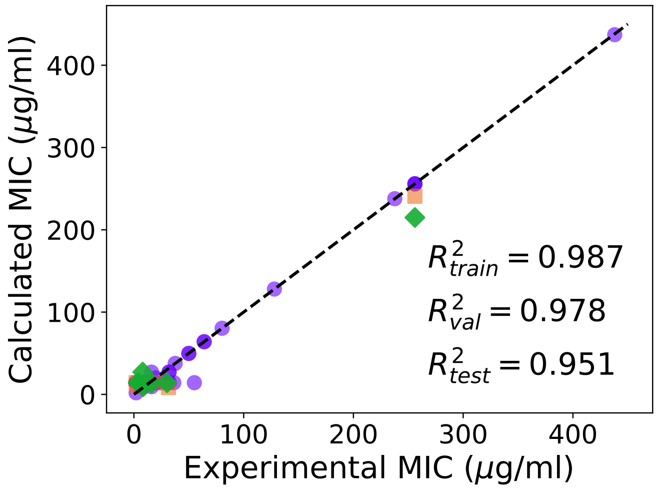

Fig F. Comparison of the experimental and calculated MIC (μg/ml) of curated AMPs on *A. baumannii* obtained from Model-2, calculated by using 6 hidden neurons. Training (purple circles), validation (orange squares) and test (green diamonds) sets are shown. The data used in the analysis for the peptides given in **Table A in S1 File**. The raw data is in **S2 File**.

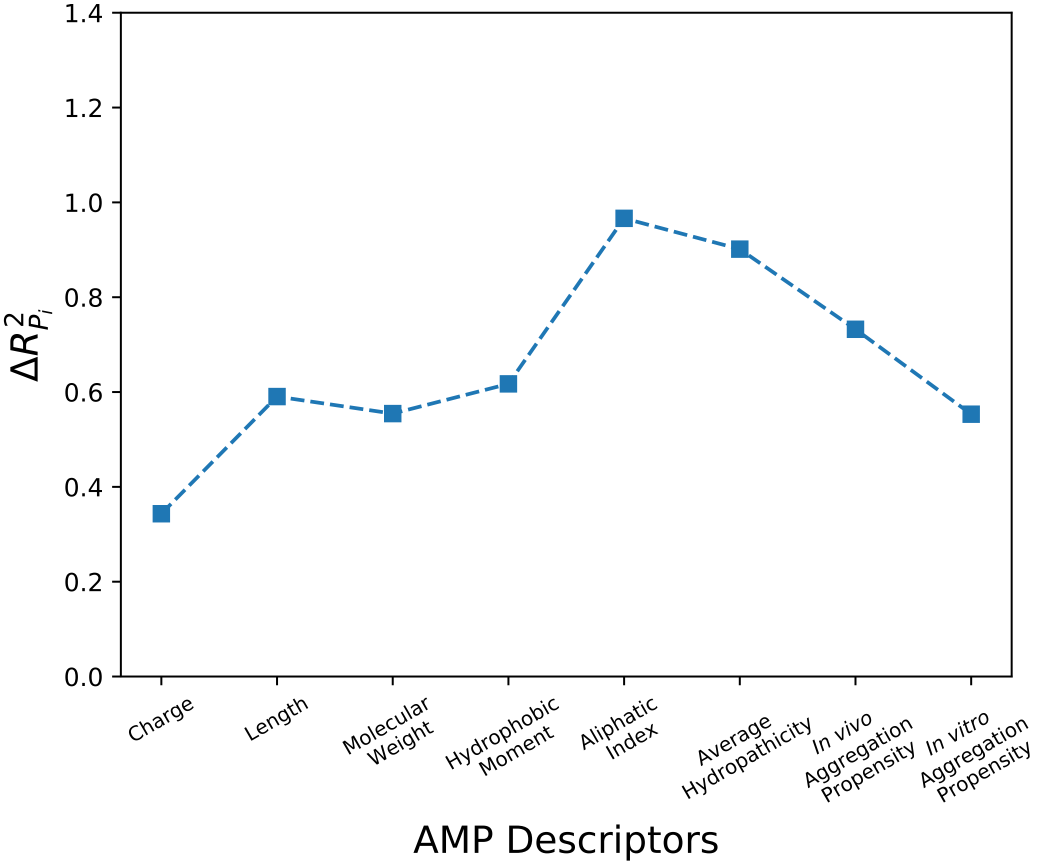

Fig G. The relative importance of the different parameters is shown for Model-2. Aliphatic index continues to be the most relevant variable, similar to **Fig 3** of the main text.

Table D. Comparison of the experimental and predicted MIC values for the MDR *A. baumannii* strains. The results were satisfactory for most of these cases. Polydim-I has extremely low values in one of the parameter used in the model (μ_H), which is not falling in the training range.

**Peptide**	**Expt. MIC** μ**g/ml**	**Model-1 MIC** μ**g/ml**	**Model-2 MIC** μ**g/ml**	**Reference**
Agelaia-MPI	39.2	76.1	9.4	[15]
Polybia-MPII	40.4	76.1	9.4	[15]
Polydim-I	> 61.1	-110.9	-172.2	[15]
Con10	70.6	74.8	-174.7	[15]
NDBP-5.8	> 37.8	76.0	9.4	[15]
LS-sarcotoxin	4	20.7	14.2	[16]
LS-stomoxyn	4	76.1	14.2	[16]
BP56	4	4.6	14.2	[17]

References

1. Vila-Farres X, De La Maria CG, López-Rojas R, Pachón J, Giralt E, Vila J. In vitro activity of several antimicrobial peptides against colistin-susceptible and colistin-resistant Acinetobacter baumannii. Clinical Microbiology and Infection. 2012;18(4):383–387.

2. Peng SY, You RI, Lai MJ, Lin NT, Chen LK, Chang KC. Highly potent antimicrobial modified peptides derived from the Acinetobacter baumannii phage endolysin LysAB2. Scientific reports. 2017;7(1):11477.

3. Conlon JM, Ahmed E, Pal T, Sonnevend A. Potent and rapid bactericidal action of alyteserin-1c and its [E4K] analog against multidrug-resistant strains of Acinetobacter baumannii. Peptides. 2010;31(10):1806–1810.

4. Jaśkiewicz M, Neubauer D, Kazor K, Bartoszewska S, Kamysz W. Antimicrobial activity of selected antimicrobial peptides against planktonic culture and biofilm of Acinetobacter baumannii. Probiotics and Antimicrobial Proteins. 2018; p. 1–8.

5. Mohamed MF, Brezden A, Mohammad H, Chmielewski J, Seleem MN. A short D-enantiomeric antimicrobial peptide with potent immunomodulatory and antibiofilm activity against multidrug-resistant Pseudomonas aeruginosa and Acinetobacter baumannii. Scientific Reports. 2017;7(1):6953.

6. Li D, Yang Y, Tian Z, Lv J, Sun F, Wang Q, et al. Synergistic antibiotic effect of looped antimicrobial peptide CLP-19 with bactericidal and bacteriostatic agents. Oncotarget. 2017;8(34):55958–55966.

7. Kohn EM, Shirley DJ, Arotsky L, Picciano AM, Ridgway Z, Urban MW, et al. Role of cationic side chains in the antimicrobial activity of C18G. Molecules. 2018;23(2):329.

8. Wiegand I, Hilpert K, Hancock RE. Agar and broth dilution methods to determine the minimal inhibitory concentration (MIC) of antimicrobial substances. Nature protocols. 2008;3(2):163.

9. Andrä J, Monreal D, de Tejada GM, Olak C, Brezesinski G, Gomez SS, et al. Rationale for the design of shortened derivatives of the NK-lysin derived antimicrobial peptide NK-2 with improved activity against Gram-negative pathogens. Journal of Biological Chemistry. 2007;.

10. Almaaytah A, Ya'u A, Abualhaijaa A, Tarazi S, Alshar'i N, Al-Balas Q. Peptide consensus sequence determination for the enhancement of the antimicrobial activity and selectivity of antimicrobial peptides. Infection and drug resistance. 2017;10:1–17.

11. Thomsen TT. Peptide Antibiotics for ESKAPE Pathogens: Past, Present and Future Perspectives of Antimicrobial Peptides for the Treatment of Serious Gram-Negative and Gram-Positive Infections. Department of Biology, Faculty of Science, University of Copenhagen; 2016.

12. Almaaytah A, Qaoud MT, Abualhaijaa A, Al-Balas Q, Alzoubi KH. Hybridization and antibiotic synergism as a tool for reducing the cytotoxicity of antimicrobial peptides. Infection and Drug Resistance. 2018;11:835–847.

13. Jayamani E, Rajamuthiah R, Larkins-Ford J, Fuchs BB, Conery AL, Vilcinskas A, et al. Insect-derived cecropins display activity against Acinetobacter baumannii in a whole-animal high-throughput Caenorhabditis elegans model. Antimicrobial agents and chemotherapy. 2015; p. 1728–1737.

14. Reller LB, Weinstein M, Jorgensen JH, Ferraro MJ. Antimicrobial susceptibility testing: a review of general principles and contemporary practices. Clinical infectious diseases. 2009;49(11):1749–1755.

15. das Neves RC, Mortari MR, Schwartz EF, Kipnis A, Junqueira-Kipnis AP. Antimicrobial and Antibiofilm Effects of Peptides from Venom of Social Wasp and Scorpion on Multidrug-Resistant Acinetobacter baumannii. Toxins. 2019;11(4):216.

16. Hirsch R, Wiesner J, Marker A, Pfeifer Y, Bauer A, Hammann PE, et al. Profiling antimicrobial peptides from the medical maggot Lucilia sericata as potential antibiotics for MDR Gram-negative bacteria. Journal of Antimicrobial Chemotherapy. 2018;74(1):96–107.

17. Di Bonaventura I, Baeriswyl S, Capecchi A, Gan BH, Jin X, Siriwardena TN, et al. An antimicrobial bicyclic peptide from chemical space against multidrug resistant Gram-negative bacteria. Chemical communications. 2018;54(40):5130–5133.
